# A Trip to Tennessee

**Published:** 1857-06

**Authors:** 


### EDITORIAL AND MISCELLANEOUS.

### A TRIP TO TENNESSEE.

It was our privilege to attend the recent celebration of the completion of the Memphis & Charleston Railroad, in the City of Memphis, from which place, we proceeded to Nashville, as a delegate to the American Medical Association, and have very rarely had altogether so pleasant a visit anywhere, as this, to the noble State of Tennessee. Memphis is rapidly becoming one, of the most important cities in the West, and with the energy, enterprise, and expanded views prevailing there, it is difficult to put a limit upon the prospects of the city; with all our hearts, we say, may they realize all and even more than they anticipate,—by the way, we hope to see the Medical College of that city, keeping pace in growth and prosperity with the other interests, and indeed we know but few points better located for a Medical School, than this, and hope soon to see the talent and science belonging to the Faculty fully rewarded. Long shall we remember the public, and especially the *private* hospitality of the City of Memphis.

As before intimated, from Memphis, we proceeded to Nashville, to attend the meeting of the American Medical Association, which convened in that city on the 5th of May; and here again, we should be ungrateful indeed, and utterly dead to all the more honorable and elevated emotions, if we had failed to be responsive to the generous, and we may say magnificent entertainment of the members of the Association by the "City of Rocks;" and upon the whole, we have returned to our homes, fully convinced that the people of Tennessee are a noble and generous hearted race, and do every thing upon a large scale; and we doubt exceedingly whether there is a place in the Union, where a more elegant and profuse hospitality can be found than in the City of Nashville; and it would appear that the Science of Medicine must be more highly appreciated there than we usually find it elsewhere, as the citizens generally seemed to vie with the medical men of the city, in bestowing distinguished attention upon the Association.

